# The trend in quality of life of Chinese population: analysis based on population health surveys from 2008 to 2020

**DOI:** 10.1186/s12889-023-15075-2

**Published:** 2023-01-24

**Authors:** Dingyao Wang, Shitong Xie, Jing Wu, Bei Sun

**Affiliations:** 1 School of Pharmaceutical Science and Technology, Tianjin University, Tianjin, 300072 China; 2 Center for Social Science Survey and Data, Tianjin University, Tianjin, China; 3 Department of Health Research Methods, Evidence, and Impact, McMaster University, Hamilton, Ontario Canada; 4 Academy of Medical Engineering and Translational Medicine, Tianjin University, Tianjin Medical University Cancer Institute and Hospital, National Clinical Research Center for Cancer, Key Laboratory of Cancer Prevention and Therapy, Tianjin, Tianjin’s Clinical Research Center for Cancer, Tianjin, 300072 China

**Keywords:** Quality of life, EQ-5D, Trend, Population health surveys, China

## Abstract

**Background:**

Quality of life (QoL) is one of the most important indicators for evaluating an individual’s overall health status. However, evidence exploring the trend in QoL of the Chinese population is still lacking. This study aimed to investigate the trend in QoL of the Chinese population measured by the EQ-5D from 2008 to 2020, as well as compare the changing trends in QoL categorized by populations with different socio-demographic characteristics.

**Methods:**

Data were obtained from the 2008, 2013, and 2020 waves of the Health Services Surveys conducted in Tianjin, China. Respondents completed the EQ-5D (EQ-5D-3L in 2008 and 2013 and EQ-5D-5L in 2020) through face-to-face interviews or self-administration. Responses of the EQ-5D-3L in 2008 and 2013 were mapped onto the EQ-5D-5L responses, and then converted to utility values using the Chinese value set. The trend in QoL was explored by comparing the percentage of any reported problems on each EQ-5D dimension and the corresponding utility values across the three waves. Subgroup analyses were performed to compare trends in utility values stratified by socio-demographic indicators. The effect of the time variable (year) on utility values was assessed by multiple linear regression analyses using the pooled data.

**Results:**

By analyzing and comparing the three waves of the data (*N* = 25,939 in the 2008 wave, *N* = 22,138 in 2013, and *N* = 19,177 in 2020), an upward trend was observed in the percentages of reporting problems on all five dimensions (*p* < 0.001), resulting in a decreasing trend in utility values (2008: 0.948, 2013: 0.942, 2020: 0.939, *p* < 0.001). Utility values declined more over time among the female, the elder, the recipients of medical assistance, the widowed, the unemployed, and respondents with primary or lower education. The effect of the year (Coef. for 2013 = − 0.009, *p* < 0.001; Coef. for 2020 = − 0.010, *p* < 0.001) confirmed the downward trend in the utility values.

**Conclusions:**

The overall QoL of the Chinese population decreased over the period from 2008 to 2020. The QoL of the disadvantaged or vulnerable populations in terms of socioeconomic characteristics declined more over time.

**Supplementary Information:**

The online version contains supplementary material available at 10.1186/s12889-023-15075-2.

## Introduction

Quality of life (QoL) is defined as individuals’ subjective evaluations of their position in life in the context of the cultural value system and in relation to their goals, expectations, standards, and concerns, which has been widely used to assess an individual’s health status [1]. This means Qol is a multi-dimensional concept that refers to individuals’ physical, mental, and social domains of well-being, as well as personal beliefs, level of independence, and their relationships with the environment [1, 2]. The QoL of the residents in a region could be measured through the health surveys for the resident population, in which the results of QoL can offer integral information on the overall situation and longitudinal trend of the residents’ health status [3, 4]. Besides, it can also provide empirical evidence for supporting healthcare decision-making [3, 4].

The QoL can be evaluated by generic preference-based measures (GPBMs), usually consisting of a health state descriptive system and a corresponding country-specific health utility value set elicited from a representative sample of the general population [5, 6]. The health utility lies on a standard scale, where the upper boundary 1 represents full health, 0 represents death, and values lower than 0 represent the health states that are deemed as worse than death. It provides a standardized score to interpret the severity of the health state [7]. The GPBMs have been increasingly used in population health surveys owing to the good performance on reliability and validity, and acceptable cognitive burden for respondents [8–10].

The EQ-5D, developed by the EuroQol Group, is one of the most commonly used GPBMs worldwide [11]. The original version, EQ-5D-3L, comprised five dimensions with three severity levels each [12]. The newly developed version, EQ-5D-5L, keeps the original five dimensions but expands the severity levels from three to five in order to allow obtaining a wider range of health state descriptions and thus improving the sensitivity of detecting changes in QoL [13]. Both versions of the EQ-5D have been validated and are widely used for assessing the QoL of the Chinese population [9, 10, 14, 15].

Several studies have been conducted to assess QoL changes over time among the general population in various countries or regions [16–21], but only two previous studies were conducted in China, including Mainland China and Hong Kong [16, 17]. The study conducted in Mainland China, which used the data from two waves of national population health surveys from 2008 to 2013, reported a slightly decreasing trend in QoL of the general population using the EQ-5D-3L [16]. Another study conducted in Hong Kong, which analyzed four waves of Hong Kong population health surveys data from 1998 to 2015, observed a V-shape in the trend of utility values (with the lowest value in 2008) using the original version of Short Form Six-Dimension (SF-6Dv1) [17]. In addition, both studies found that disparities in QoL across different demographic and socioeconomic subgroups are increasing in China [16, 17]. However, to the best of our knowledge, there is currently no study that has explored the long-term trend in QoL of a large sample of the Chinese population, especially in the past decade. Besides, in recent years, whether the inequalities in the QoL of populations with different socio-demographic characteristics keep enlarging is still unclear.

Therefore, this study aimed to explore the long-term trend in QoL of the Chinese population measured by the EQ-5D from 2008 to 2020, as well as compare the changing trends in QoL reflected by people with different socio-demographic characteristics.

## Methods

### Data sources and study sample

This study was a secondary data analysis. Data used in this study were obtained from Tianjin Health Services Surveys (TJHSS). Tianjin is one of the four municipalities in China, with nearly 14 million permanent residents [22]. The TJHSS has been conducted six times since 1993 (in 1993, 1998, 2003, 2008, 2013, and 2020) by Tianjin Municipal Health Commission [23]. The data for the 2008, 2013, and 2020 waves were used in this study since the GBPM was first involved in the 2008 wave. The EQ-5D-3L was employed in the 2008 and 2013 waves, while the EQ-5D-5L was used in the 2020 wave of TJHSS.

A multi-stage, stratified cluster random sampling strategy was used in all 16 districts of Tianjin to recruit respondents for the TJHSS [23]. Five subdistricts /townships in each district were first randomly selected. The second stage narrowed the areas down to two communities/villages within each subdistrict/township. Approximately 60 households from each community/village were finally included in the TJHSS. All family members registered in each household were asked to participate in the survey.

Data from the TJHSS were collected mainly through paper-based face-to-face household interviews (about 40% of respondents self-reported due to the COVID-19 administrative policy in the 2020 wave). First, after obtaining informed consent from each respondent, the respondent who was the most familiar with their family situations was asked to answer some basic questions, including the annual household medication expenditures and the distance to the closest healthcare institute from home. Second, all the eligible respondents completed social-demographic questions on gender, ethnicity, date of birth, health insurance coverage, medical assistance, marital status, education level, and employment status. Third, respondents aged ≥15 years were asked to complete the EQ-5D and health-related questions, including the presence of health examination in the past 12 months, presence of hypertension, diabetes, or other chronic diseases, number of illnesses in 2 weeks, and the presence of hospitalizations in 12 months. Forth, several questions, i.e., the delivery place, the number of children for female respondents (aged 15 to 64 years), and the presence of vaccination certificates, height, and weight at birth for adolescent respondents (aged < 5 years), were also asked if eligible. Detailed descriptions of sampling and data collection can be found elsewhere [23, 24].

For this study, data collected in the second and third parts of the 2008, 2013, and 2020 waves of TJHSS were used. Respondents aged < 18 years were excluded from this current analysis since the EQ-5D is recommended to be used among adult respondents by the EQ-5D user guide [25]. Respondents included in this study were also required to meet the following criteria: (i) had no missing values for the EQ-5D; and (ii) had no missing values for other variables included in the current analyses, including socio-demographic characteristics and health indicators.

### Instruments

The EQ-5D-3L comprises five dimensions, namely, mobility, self-care, usual activities, pain/discomfort, and anxiety/depression, with each containing three response levels (no, moderate, and extreme problems). The EQ-5D-3L describes a total of 243 (=3^5^) health states, with 11,111 being the best health state and 33,333 the worst. The Chinese utility value set for the EQ-5D-3L was generated based on the time trade-off (TTO) approach, with the range of − 0.149 (33333) to 1 (11111) [26].

The EQ-5D-5L comprises the same five dimensions as the EQ-5D-3L but has five levels of severity (no, slight, moderate, severe, and extreme problems) for each dimension. The EQ-5D-5L defines a total of 3125 (=5^5^) health states, with 11,111 being the best health state and 55,555 the worst. The Chinese utility value set for the EQ-5D-5L was developed using the TTO approach, with the range of utility value from − 0.391 (55555) to 1 (11111) [27].

### Mapping between EQ-5D-3L and EQ-5D-5L

In order to reduce the impact of the instrument inconsistency and make the QoL results from the three waves of data more comparable, the utility derived from the three waves need to be aligned into the same instrument. Given no mapping algorithms between these two instruments are available in China, the UK response mapping algorithm was used in this study as the main analysis [28]. The probabilities of mapping EQ-5D-3L responses to EQ-5D-5L responses were obtained based on the UK response mapping algorithm [28]. Three methods of calculating the utility values for the mapped responses were used according to the literature [29, 30], including the expected-utility method, the Monte Carlo simulation method, and the most-likely probability method. The expected-utility method was chosen in the main analysis due to its better predictive ability [29, 30], and the other two methods were used for the sensitivity analysis.

Besides, the Decision Support Unit (DSU) mapping approach developed by the National Institute for Health and Care Excellence (NICE) was also used to convert the EQ-5D-5L utility values into the EQ-5D-3L utility values [31]. This additional analysis was also conducted to validate the results obtained from the main analysis. The DSU model predictions were calculated using the Stata command EQ 5DMAP with the Model = *EQGcopula* option [32].

### Statistical analysis

Descriptive summary statistics (mean, standard deviation [SD], frequency, and proportion) were used to describe the characteristics of respondents included in the three waves of TJHSS. One-way analyses of variance were performed for continuous variables, and Chi-square (χ^2^) tests were used for categorical variables.

The trajectory of each dimension of the EQ-5D was examined separately (proportion of reporting any health problems on each dimension) by line charts to explore which dimensions were driving the overall trend. Besides, age-standardized proportions of reported problems on each dimension were examined in the current analyses. The proportions were tested by Chi-square (χ^2^) tests. The trend in utility values was reported using bar charts and tested by the Kruskal-Wallis H tests. The importance of changes in the utility values was also estimated using effect sizes (ES), which were calculated as the difference between the highest and the lowest utility values among the three waves divided by the pooled standard deviation [4]. The general guideline defines ES of 0.8 as large, 0.5 to 0.79 as moderate, and 0.2 to 0.49 as small [33], and moderate effect sizes (≥ 0.5) are usually considered as a difference with clinical meaning [34]. Furthermore, the age-standardized utility values in three waves were also examined.

Subgroup analyses were performed to compare the changing trends in utility values reflected by the respondents with various characteristics. Kruskal-Wallis H tests were applied to compare the utility values between subgroups. The trend in QoL stratified by the key social-demographic variables, including gender, age groups, recipients of medical assistance, marital status, education, and employment status, was performed by the line chart for the within-group comparisons.

Multiple linear regression models with the robust standard error were established to explore the changing trend of the QoL using the pooled data after adjusting for other characteristics (e.g., gender, age, and education level) [17, 35]. A series of dummy variables were set to distinguish the three waves of the data. Multicollinearity between covariates was checked by the variation inflation factors (VIF). The goodness-of-fit of the regression models was measured by the coefficient of determination (R^2^) [35].

All the statistical analyses were performed using STATA 15.0 (StataCorp LLC, College Station, TX, USA). All reported statistical tests were performed two-sided with a significance level of 0.05. The protocol and data collection process of the Tianjin Health Service Survey were approved by the Institutional Review Board of Tianjin Municipal Health Commission (2008: No. [2008]225; 2013: No. [2013]23; No. [2020]549). Besides, the protocol of this study was approved by the Academic Ethics Committee at Tianjin University (No. THUE-2021-168).

## Results

Respondents under 18 years (4263, 3076, and 3754 in the 2008, 2013, and 2020 waves, respectively) and respondents who returned questionnaires with missing values (993, 72, and 1220 in the 2008, 2013, and 2020 waves, respectively) were excluded from the current analyses. A total of 25,939 respondents in the 2008 wave, 22,138 respondents in the 2013 wave, and 19,177 respondents in the 2020 wave were included in this study.

### Characteristics of respondents

As presented in Table 1, the distributions of gender and ethnic group were similar among the three waves (*p* > 0.05). Compared with the 2008 and 2013 waves, respondents in the 2020 wave were older (*p* < 0.001), had a higher married proportion (*p* < 0.001), and reported a higher level of education (*p* < 0.001), but had a lower employed proportion (*p* < 0.001). There was an increase (*p* < 0.001) in the proportion of respondents with hypertension or diabetes, as 35.5% had hypertension (17.9% in 2008, 25.2% in 2013), and 13.5% had diabetes in 2020 (4.2% in 2008, 8.1% in 2013) (Table 1).

**Table 1:** Characteristics of study respondents, 2008–2020

Characteristics	2008 (***N*** = 25,939)	2013 (***N*** = 22,138)	2020 (***N*** = 19,177)	***p*** value
	N (%)	N (%)	N (%)	
**Gender**				0.232
Male	12,600 (48.6%)	10,748 (48.6%)	9453 (49.3%)	
Female	13,339 (51.4%)	11,390 (51.4%)	9724 (50.7%)	
**Ethnic group**				0.317
Han Chinese	25,464 (98.2%)	21,753 (98.3%)	18,862 (98.4%)	
Others	475 (1.8%)	385 (1.7%)	315 (1.6%)	
**Age (Mean, [SD])**	49.2 (17.1)	51.7 (16.8)	55.2 (16.2)	< 0.001
**Age group (years)**				< 0.001
18–29	4329 (16.7%)	3003 (13.6%)	1655 (8.6%)	
30–39	3399 (13.1%)	2695 (12.2%)	2319 (12.1%)	
40–49	4864 (18.8%)	3460 (15.6%)	2317 (12.1%)	
50–59	6011 (23.2%)	4976 (22.5%)	3615 (18.9%)	
60–69	3747 (14.4%)	4884 (22.1%)	5830 (30.4%)	
≥ 70	3589 (13.8%)	3120 (14.1%)	3441 (17.9%)	
**Commercial medical insurance**				< 0.001
Yes	1240 (4.8%)	964 (4.4%)	1918 (10.0%)	
No	24,699 (95.2%)	21,174 (95.6%)	17,259 (90.0%)	
**Recipients of medical assistance**				< 0.001
Yes	305 (1.2%)	209 (0.9%)	391 (2.0%)	
No	25,634 (98.8%)	21,929 (99.1%)	18,786 (98.0%)	
**Marital status**				< 0.001
Unmarried	3386 (13.1%)	2257 (10.2%)	1736 (9.1%)	
Married	20,311 (78.3%)	17,990 (81.3%)	15,833 (82.6%)	
Widowed	1970 (7.6%)	1617 (7.3%)	1285 (6.7%)	
Divorced	272 (1.0%)	274 (1.2%)	323 (1.7%)	
**Education**				< 0.001
Primary or below	7792 (30.0%)	5510 (24.9%)	4385 (22.9%)	
Junior high school	9719 (37.5%)	8587 (38.8%)	7365 (38.4%)	
Senior high school	5309 (20.5%)	4584 (20.7%)	3923 (20.5%)	
College or above	3119 (12.0%)	3457 (15.6%)	3504 (18.3%)	
**Employment status**				< 0.001
Employed	12,473 (48.1%)	10,973 (49.6%)	7035 (36.7%)	
Retired	6161 (23.8%)	5995 (27.1%)	6279 (32.7%)	
Student	1024 (3.9%)	514 (2.3%)	429 (2.2%)	
Unemployed	6281 (24.2%)	4656 (21.0%)	5434 (28.3%)	
**Hypertension**				< 0.001
Yes	4653 (17.9%)	5581 (25.2%)	6806 (35.5%)	
No	21,286 (82.1%)	16,557 (74.8%)	12,371 (64.5%)	
**Diabetes**				< 0.001
Yes	1096 (4.2%)	1785 (8.1%)	2586 (13.5%)	
No	24,843 (95.8%)	20,353 (91.9%)	16,591 (86.5%)	
**Other chronic diseases**				< 0.001
Yes	3945 (15.2%)	2345 (10.6%)	1082 (5.6%)	
No	21,994 (84.8%)	19,793 (89.4%)	18,095 (94.4%)	
**Number of illnesses in 2 weeks**				< 0.001
0	24,570 (94.7%)	20,163 (91.1%)	17,523 (91.4%)	
1	901 (3.5%)	1761 (8.0%)	1377 (7.2%)	
2 or more	468 (1.8%)	214 (1.0%)	277 (1.4%)	
**Hospitalizations in 12 months**				< 0.001
Yes	1246 (4.8%)	1173 (5.3%)	733 (3.8%)	
No	24,693 (95.2%)	20,965 (94.7%)	18,444 (96.2%)	

*Note*: Chi-square tests were performed to identify statistically significant differences of the respondents’ characteristics in three waves of TJHSS for categorical variables, while one-way analyses of variance test were performed for continuous variables

*Abbreviation*: *SD* standard deviation

### Trends in quality of life

**Fig. 1:**
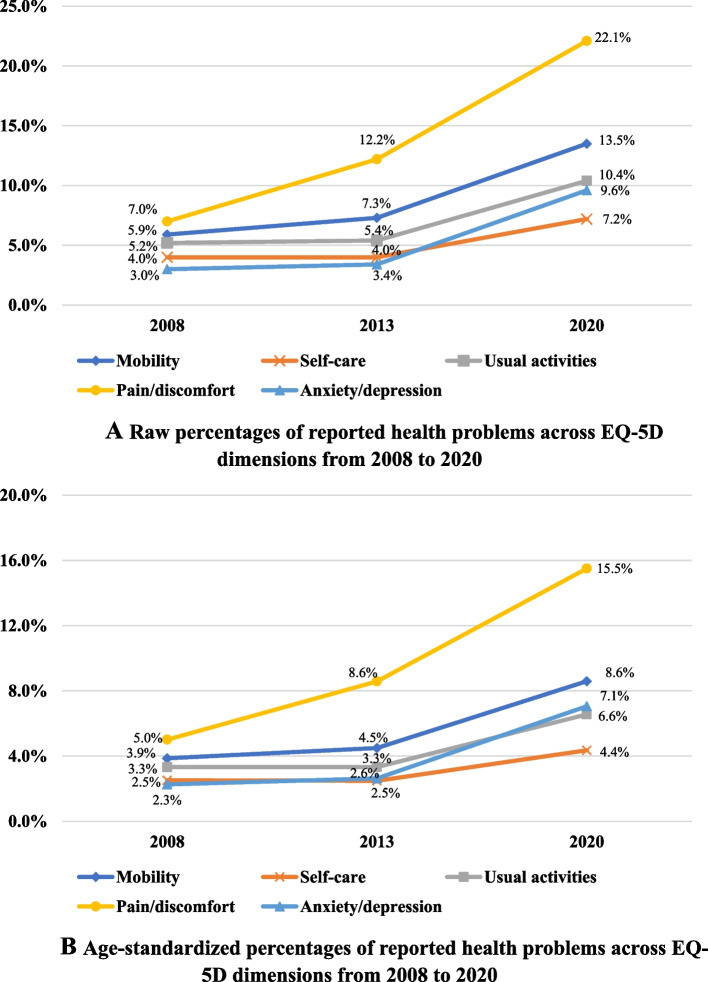
**A** Raw percentages of reported health problems across EQ-5D dimensions from 2008 to 2020. **B** Age-standardized percentages of reported health problems across EQ-5D dimensions from 2008 to 2020. Note: The EQ-5D-3L was used in 2008 and 2013, while the EQ-5D-5L was used in 2020. Significant differences were found in the raw and age-standardized percentage of reported any health problems in each dimension from 2008 to 2020 by Chi-square (χ2) tests (*p* < 0.001)

89.6% (*N* = 23,231), 84.0% (*N* = 18,603), and 72.8% (*N* = 13,961) of respondents reported full health state (no problems on all EQ-5D dimensions) in the 2008, 2013, and 2020 waves, respectively. The raw proportions of respondents who reported any health problems on each EQ-5D dimension are shown in Fig. 1A. The highest proportion of reporting any problems was always observed in the pain/discomfort dimension (2008: 7.0%, 2013: 12.2%, 2020: 22.1%), followed by mobility (2008: 5.9%, 2013: 7.3%, 2020: 13.5%) and usual activities (2008: 5.2%, 2013: 5.4%, 2020: 10.4%). Notably, the anxiety/depression dimension was consistently the least reported health problem in 2008 (3.0%) and 2013 (3.4%), but it surpassed the self-care dimension and reached 9.6% in 2020. Besides, the results of age-standardized proportions of reported problems on each EQ-5D dimension show a consistent trend with the raw results, but the absolute values of proportions become smaller in all three waves (Fig. 1B).

**Fig. 2:**
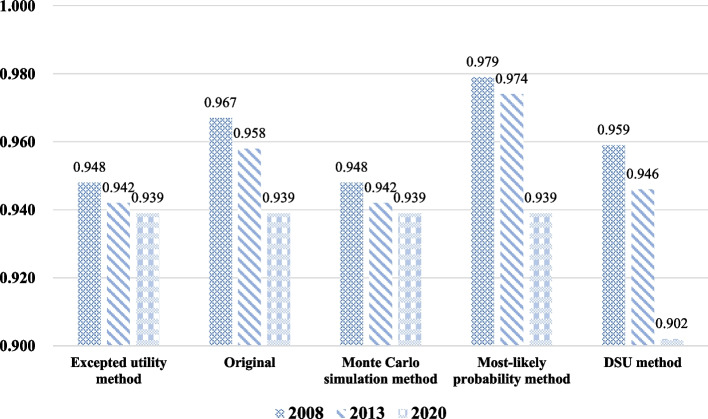
Trend in health utility values of study respondents from 2008 to 2020. Note: Significant differences were found in health utility values across the three waves of data by Kruskal-Wallis H tests (*p* < 0.001)

Overall, the mean (SD) raw utility values were 0.967 (0.122) for the 2008 wave and 0.958 (0.125) for 2013 using the EQ-5D-3L, and 0.939 (0.168) in the 2020 wave using the EQ-5D-5L (Table S1 in Supplementary Information). After age-standardization, the mean utility values for respondents in the three waves were 0.981, 0.975, and 0.960 (*p* < 0.001). Besides, after mapping, there was still a significantly decreasing trend in the mean utility values (0.948 in 2008 vs. 0.942 in 2013 vs. 0.939 in 2020, *p* < 0.001, ES = 0.070), but the gap between the three waves narrowed (Fig. 2). This significant downward trend in utility values was also observed in the sensitivity analysis by using the Monte Carlo simulation method, the most-likely probability method, and the DSU method (*p* < 0.001) (Fig. 2).

### Subgroup analyses

**Fig. 3:**
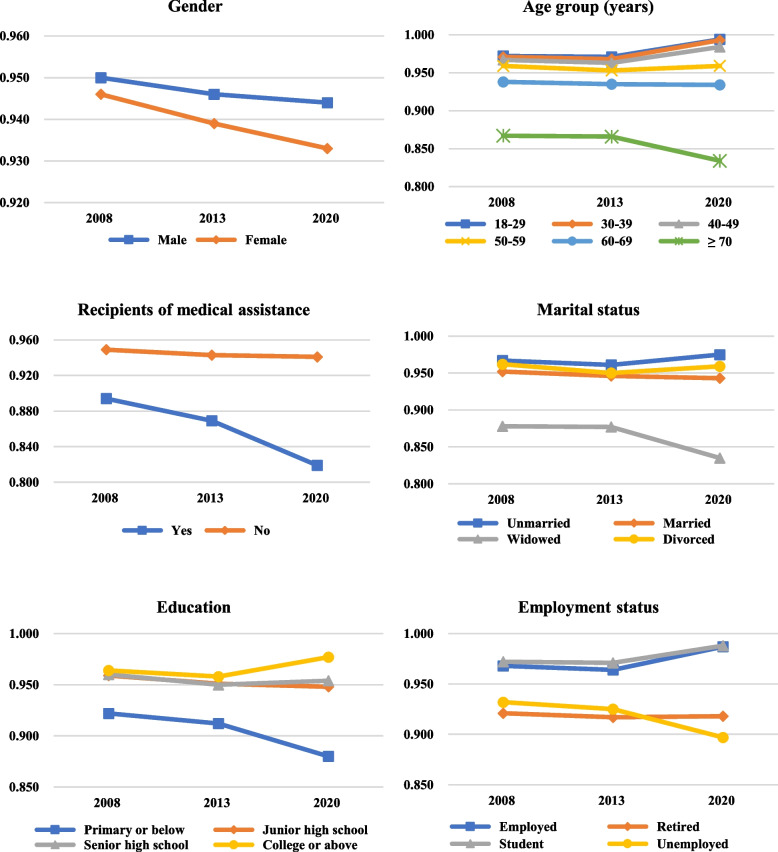
Trend in health utility values of study respondents stratified by the main socio-demographic characteristics from 2008 to 2020. Note: The EQ-5D-3L responses were mapped to EQ-5D-5L responses by the UK response mapping algorithm [28] and then converted to utility values for the mapped responses using the excepted-utility method [29]

Table 2 describes the utility values stratified by characteristics of respondents from 2008 to 2020. Although an upward trend was observed in a few subgroups with better socioeconomic status (e.g., younger, employed respondents and respondents who had higher educational attainments), the trend in utility values was consistently decreased in most subgroups (*p* < 0.05, ES ranging from 0.016 to 0.535). Utility values reported by the female, the elderly, the recipients of medical assistance, the widowed, the unemployed, and respondents with primary or lower education declined more, with generally larger values of ES (Fig. 3). The results of the sensitivity analysis were also consistent with the results described above (results not shown).

**Table 2:** Health utility values stratified by characteristics of respondents, 2008–2020

Characteristics	Mean health utility values ^**a**^ (SD)			ES ^**b**^ (95% CI)	***p*** value^*^
	2008 (***N*** = 25,939)	2013 (***N*** = 22,138)	2020 (***N*** = 19,177)		
**Total**	0.948 (0.102)	0.942 (0.103)	0.939 (0.168)	0.070 (0.051, 0.089)	< 0.001
**Gender**					
Male	0.950 (0.102)	0.946 (0.097)	0.944 (0.167)	0.046 (0.020, 0.073)	< 0.001
Female	0.946 (0.101)	0.939 (0.108)	0.933 (0.169)	0.093 (0.067, 0.120)	< 0.001
**Ethnic group**					
Han Chinese	0.948 (0.101)	0.942 (0.103)	0.939 (0.168)	0.070 (0.051, 0.089)	< 0.001
Others	0.939 (0.116)	0.937 (0.100)	0.930 (0.168)	0.068 (−0.074, 0.210)	< 0.001
**Age group (years)**					
18–29	0.972 (0.026)	0.971 (0.031)	0.994 (0.067)	0.483 (0.422, 0.544)	< 0.001
30–39	0.971 (0.029)	0.968 (0.044)	0.993 (0.050)	0.535 (0.479, 0.592)	< 0.001
40–49	0.967 (0.046)	0.963 (0.051)	0.984 (0.078)	0.335 (0.282, 0.388)	< 0.001
50–59	0.959 (0.065)	0.953 (0.073)	0.959 (0.129)	0.057 (0.014, 0.100)	< 0.001
60–69	0.938 (0.117)	0.935 (0.106)	0.934 (0.161)	0.027 (−0.014, 0.068)	< 0.001
≥ 70	0.867 (0.201)	0.866 (0.189)	0.834 (0.267)	0.140 (0.093, 0.186)	< 0.001
**Commercial medical insurance**					
Yes	0.967 (0.043)	0.958 (0.061)	0.972 (0.108)	0.143 (0.066, 0.221)	< 0.001
No	0.947 (0.103)	0.942 (0.104)	0.935 (0.173)	0.089 (0.069, 0.108)	< 0.001
**Recipients of medical assistance**					
Yes	0.894 (0.154)	0.869 (0.181)	0.819 (0.317)	0.290 (0.140, 0.441)	< 0.001
No	0.949 (0.101)	0.943 (0.102)	0.941 (0.162)	0.058 (0.039, 0.077)	< 0.001
**Marital status**					
Unmarried	0.967 (0.052)	0.961 (0.069)	0.975 (0.119)	0.146 (0.084, 0.209)	< 0.001
Married	0.952 (0.094)	0.946 (0.095)	0.943 (0.160)	0.070 (0.049, 0.091)	< 0.001
Widowed	0.878 (0.188)	0.877 (0.180)	0.835 (0.264)	0.195 (0.124, 0.265)	< 0.001
Divorced	0.962 (0.041)	0.950 (0.070)	0.959 (0.138)	0.200 (0.032, 0.368)	< 0.001
**Education**					
Primary or below	0.922 (0.147)	0.912 (0.146)	0.880 (0.233)	0.224 (0.187, 0.262)	< 0.001
Junior high school	0.959 (0.075)	0.951 (0.086)	0.948 (0.150)	0.097 (0.067, 0.127)	< 0.001
Senior high school	0.960 (0.072)	0.950 (0.084)	0.954 (0.140)	0.053 (0.012, 0.094)	< 0.001
College or above	0.964 (0.056)	0.958 (0.066)	0.977 (0.103)	0.222 (0.175, 0.269)	< 0.001
**Employment status**					
Employed	0.968 (0.041)	0.964 (0.048)	0.987 (0.069)	0.392 (0.362, 0.422)	< 0.001
Retired	0.921 (0.146)	0.917 (0.136)	0.918 (0.184)	0.029 (−0.006, 0.065)	< 0.001
Student	0.972 (0.023)	0.971 (0.027)	0.988 (0.075)	0.311 (0.182, 0.440)	< 0.001
Unemployed	0.932 (0.129)	0.925 (0.127)	0.897 (0.220)	0.197 (0.161, 0.233)	< 0.001
**Hypertension**					
Yes	0.902 (0.163)	0.909 (0.142)	0.894 (0.216)	0.078 (0.043, 0.114)	< 0.001
No	0.959 (0.079)	0.954 (0.083)	0.963 (0.128)	0.094 (0.071, 0.118)	< 0.001
**Diabetes**					
Yes	0.889 (0.180)	0.894 (0.163)	0.885 (0.224)	0.047 (−0.013, 0.107)	< 0.001
No	0.951 (0.096)	0.947 (0.095)	0.947 (0.156)	0.029 (0.009, 0.049)	< 0.001
**Other chronic diseases**					
Yes	0.869 (0.194)	0.862 (0.183)	0.810 (0.289)	0.270 (0.202, 0.337)	< 0.001
No	0.963 (0.064)	0.952 (0.084)	0.947 (0.154)	0.140 (0.120, 0.160)	< 0.001
**Number of illnesses in 2 weeks**					
0	0.951 (0.096)	0.950 (0.087)	0.944 (0.160)	0.052 (0.033, 0.072)	< 0.001
1	0.905 (0.165)	0.868 (0.186)	0.891 (0.217)	0.211 (0.131, 0.292)	< 0.001
2 or more	0.894 (0.165)	0.829 (0.205)	0.837 (0.284)	0.365 (0.202, 0.528)	< 0.001
**Hospitalizations in 12 months**					
Yes	0.857 (0.223)	0.871 (0.188)	0.803 (0.304)	0.207 (0.116, 0.299)	< 0.001
No	0.953 (0.089)	0.946 (0.095)	0.944 (0.158)	0.016 (−0.004, 0.036)	0.015

^*^ Significant changes in health utility values (*p* < 0.05) were found from 2008 to 2020 in all independent variables by Kruskal-Wallis H tests

^a^ The EQ-5D-3L responses were mapped to EQ-5D-5L responses by the UK response mapping algorithm [28] and then converted to utility values for the mapped responses using the excepted-utility method [29]

^b^ Effect sizes were calculated as the difference between the mean utility of two groups divided by the pooled standard deviation and effect sizes of 0.8 are defined as large, 0.5 to 0.79 as moderate, and 0.2 to 0.49 as small

Abbreviation: 95% CI, 95% confidence interval; ES, effect sizes, SD, standard deviation

### Regression analyses

Table 3 presents the effects of time on QoL across the three waves of data by conducting multiple linear regression models. No multicollinearity was observed in all variables included in the model (VIF < 10). The trend in utility values remained decreased (*β*_2013_ = − 0.009, *p* < 0.001; *β*_2020_ = − 0.010, *p* < 0.001) after controlling for other independent variables (e.g., gender, age, and education level) in the main analysis. This trend was also confirmed by the sensitivity analysis (Table 3). The effects of the other independent variables on utility values in the main analyses are presented in Table S2 in the Supplementary Information.

**Table 3:** Effects of the time variable on health utility values in the multiple linear regression analyses

Time variable	Health utility values (***N*** = 67,254)			
	β	95% CI	***p*** value ^*^	R^**2**^
**Excepted utility method** ^**a**^				
Year (vs. 2008)				
2013	−0.009	(−0.012, − 0.007)	**< 0.001**	0.170
2020	−0.010	(− 0.012, − 0.009)	**< 0.001**	
**Original**				
Year (vs. 2008)				
2013	−0.014	(−0.016, − 0.012)	**< 0.001**	0.193
2020	−0.028	(− 0.031, − 0.026)	**< 0.001**	
**Monte Carlo simulation method** ^**a**^				
Year (vs. 2008)				
2013	−0.009	(−0.012, − 0.006)	**< 0.001**	0.159
2020	−0.010	(− 0.012, − 0.008)	**< 0.001**	
**Most-likely probability method** ^**a**^				
Year (vs. 2008)				
2013	−0.008	(−0.010, − 0.007)	**< 0.001**	0.165
2020	−0.040	(− 0.042, − 0.037)	**< 0.001**	
**DSU method** ^**b**^				
Year (vs. 2008)				
2013	−0.016	(−0.019, − 0.013)	**< 0.001**	0.150
2020	−0.058	(− 0.062, − 0.055)	**< 0.001**	

^*^ The *p*-value in bold formatting represents significant in multivariable linear regression model at 0.05 level

^a^ The EQ-5D-3L responses were mapped to EQ-5D-5L responses by the UK response mapping algorithm [28] and then converted to utility values for the mapped responses using the excepted-utility method [29]

^b^ The responses of respondents in 2020 elicited from the EQ-5D-5L were indirectly mapped to the EQ-5D-3L by the DSU method [31]

*Abbreviation*: *95% CI* 95% confidence interval; DSU, Decision Support Unit

## Discussion

This study found that the trend in QoL among the Chinese population was significantly declining from 2008 to 2020. The QoL of the respondents that were disadvantaged or vulnerable in terms of socioeconomic characteristics declined more over time. It is worth noting that the changes in utility values were small (ES ranging from 0.016 to 0.535), and all sub-groups (except the aged 30–39 sub-group) failed to reach the threshold of clinical importance (ES ≥ 0.5) in this study. However, considering the continuous impact of the pandemic and the increasing social competitive pressure, the results of this study can serve as an essential indicator of changes in the health of residents with the rapid development of society and economy in China. Moreover, this study provided empirical population-based evidence to inform policymakers to make suitable policy planning and better healthcare resource allocation during the years of the pandemic, which is essential for maintaining or improving the health status of the residents.

A monotonically increasing trend in the proportion of reported problems was observed in all EQ-5D dimensions, resulting in the percentage of respondents who reported a full health state declining from 89.6 to 72.8% during the past decade. The driving factors might be that the pain/discomfort (raw proportion: from 7.0 to 22.1%, age-standardized proportion: from 5.0 to 15.5%), the mobility (raw proportion: from 5.9 to 13.5%, age-standardized proportion: from 3.9 to 8.6%), and the anxiety/depression dimensions (raw proportion: from 3.0 to 9.6%, age-standardized proportion: from 2.3 to 7.1%) showed a unignorably upward trend in the percentage of reported problems, especially over the period from 2013 to 2020. One possible explanation might be that the data from the third wave is from the year 2020, which is one of the years of the Covid-19 pandemic. Previous studies have shown that the pandemic and its measures of isolation may affect both the physical and mental health of the population [36–38], which may also be the reason for the increasing proportion of reported health problems in the current study. However, further studies are needed to verify this possibility. A similar finding was also reported in one study conducted in Norway [19], which analyzed two waves of data from 2007 to 2016. However, the study conducted in Mainland China from 2008 to 2013 found that only the pain/discomfort (from 9.3 to 12.6%) dimension showed an increasing trend in the percentage of reported problems [16]. This trend is consistent with this study over the period from 2008 to 2013 but different from that reported from 2013 to 2020. One potential explanation could be that, with the rapid economic and social development in China, the general public might encounter more mental stress from their families, job careers, and social environment. Thus, they pay more attention to their mental health, especially in recent years [39, 40]. Besides, as mentioned above, the measures of isolation due to the Covid-19 pandemic might also be an influencing factor [36–38].

A significantly decreasing trend in the utility values of the respondents was observed in this study. This finding (from 0.948 to 0.939) is similar to previous studies conducted in the UK (from 0.825 to 0.818) [18] and Australia (specific data not displayed) [20]. Besides, a previous study conducted in Mainland China also found a significantly downward trend in the QoL of the Chinese population over the period from 2008 to 2013 [16]. However, a previous study conducted in Hong Kong reported a V-shape trend in the utility values across four waves of data from 1998 to 2015 (range: 0.81 to 0.87, with the lowest value in 2008) [17]. An increasing trend (range: 0.84 to 0.90) was also found in a previous study conducted in Norway, with a study period from 2007 to 2016 [19]. A possible explanation could be that the proportion of female respondents was higher in the 2008 wave in the Hong Kong study (2008: 61.7%, other waves: 52.2–52.9%) and in the 2007 wave in the Norway study (2007: 53.3%, 2016: 52.5%), as female respondents usually reported lower utility values than male respondents [10, 18].

It is worth noting that the utility values reported by respondents in the current study were higher than that of the general population in some other studies measured by the EQ-5D (range: 0.81 to 0.90) [18–20]. One of the possible reasons is that the Chinese population is more unwilling to report health problems than the Western population due to cultural tradition [41]. However, the utility values reported in this study (0.939 to 0.949) were relatively close to the population norm for EQ-5D-5L (0.946) in China [10].

The utility values of the disadvantaged or vulnerable populations declined more over the study period from 2008 to 2020, which is consistent with the previous studies conducted in Mainland China [16], Hong Kong [17] and the UK [18]. Specifically, the utility value of female respondents declined by 0.013 (from 0.946 to 0.933, ES = 0.093), while the male respondents only declined by 0.006 (from 0.950 to 0.944, ES = 0.046). Similar findings could also be observed in other subgroups, including the elder (− 0.033 [≥70 years, ES = 0.140] vs. + 0.022 to − 0.004 [other age groups, ES: 0.027 ~ 0.535]), the recipients of medical assistance (− 0.075 [ES = 0.290] vs. -0.008 [ES = 0.058]), the widowed (− 0.043 [ES = 0.195] vs. + 0.008 to − 0.009 [other marital status groups, ES: 0.070 ~ 0.200]), the unemployed (− 0.035 [ES = 0.197] vs. + 0.016 to − 0.003 [other employment status groups, ES: 0.029 ~ 0.392]) and people with primary or lower education (− 0.042 [ES = 0.224] vs. + 0.013 to − 0.011 [other education groups, ES: 0.053 ~ 0.222]). This could be partly attributed to the issue of health inequality, which is broadly existing worldwide [42]. Previous studies showed that one possible factor inducing health inequality was poverty, and it was found that the low-income class may go through relatively more unmet healthcare needs than the other income classes [43–45]. Women were at risk of entering poverty and unmet healthcare needs in previous studies, so as did the elder, the widowed, and the unemployed [34, 43]. Given the potential impact of the Covid-19 pandemic, these disadvantaged populations in the current analyses may face a greater risk of entering poverty in 2020. Consequently, disadvantaged populations may have less access to some essential healthcare resources due to a heavier financial burden when encountering severe diseases [42, 46]. Furthermore, the QoL of the disadvantaged populations would be impaired more, and the disparities of utility values may keep enlarging [16–18]. Future research taking account of the income variable is required to better understand the association between poverty and the changing trend of QoL.

An opposite upward trend in the utility values was detected in a few subgroups, i.e., respondents younger than 50 years, unmarried and employed. This can be partly explained by the fact that the range of the mapped EQ-5D-5L utility values were − 0.092 and 0.974 (theoretical range: − 0.391 to 1) using the expected-utility method [29, 30]. Given over 70% of respondents were in a full health state, it could be concluded that the mean EQ-5D-5L utility values were slightly underestimated in the 2008 and 2013 waves (similar results for the Monte Carlo simulation method). However, this upward trend was negligible when using the other two mapping methods (results not shown).

The findings of this study are subject to some limitations. First, all respondents were recruited in one city, and the proportions of the respondents aged 65 years or above (2008: 19.9%, 2013: 23.5%, 2020: 32.9%) in the current study were higher than those in the Chinese general population (2010: 8.9%, 2020: 13.5%) [47, 48]. This may have an impact on the generalizability of the study results. Second, both face-to-face interviews and self-reports were used in the 2020 TJHSS to collect data, which may have an unobserved impact on the results of the current analyses to some extent. Third, the data from the third wave is from the year 2020, which is one of the years of the Covid-19 pandemic. However, there were no variables related to Covid-19 in TJHSS, and the impact of the pandemic on the Qol of the population could not be captured in this study. Future studies are required to explore the correlation between the pandemic and the decrease in QoL. Finally, the transition probabilities used to map between the EQ-5D-3L and the EQ-5D-5L were drawn from respondents in European countries. Given the potential cultural differences in health preference [49], the transition probabilities may not be ideal for the Chinese population. This is, however, currently the only official transition probability matrix available from the EuroQol Group. Future studies are required to develop the mapping algorithms between the two instruments among the Chinese population.

## Conclusion

The decreasing trend in the QoL of the Chinese population was observed over the period from 2008 to 2020. The QoL of the female, the elder, the recipients of medical assistance, the widowed, the unemployed, and respondents with primary or lower education declined more over time. Further studies are required to explore the underlining reasons, and the government should strengthen the community primary care system and provide more psychological counseling services to improve both the physical and mental health of the disadvantaged or vulnerable in terms of socioeconomic characteristics.

## Supplementary Information

**Additional file 1: Table S1.** Descriptive statistics of the health utility values, 2008–2020. **Table S2.** Multiple linear regression analyses on health utility values (Polled).

## Data Availability

The ownership of the datasets used and/or analyzed during the current study belongs to Tianjin Municipal Health Commission. The individual-level data is not publicly available. Data used in this study are however available from the corresponding author upon reasonable request.

## Abbreviations

DSU: Decision Support Unit
ES: Effect sizes
EQ-5D-3L: EQ-5D 3-level version
EQ-5D-5L: EQ-5D 5-level version
GBPMs: Generic preference-based measures
QoL: Quality of life
NICE: National Institute for Health and Care Excellence
SD: Standard deviations
SF-6Dv1: The original version of Short Form Six-Dimension
TJHSS: Tianjin Health Services Surveys
TTO: Time trade-off
VIF: Variation inflation factors
