# Supplementary material for: The trend in quality of life of Chinese population: analysis based on population health surveys from 2008 to 2020
Source: BMC Public Health. 2023 Jan 24;23:167. doi: 10.1186/s12889-023-15075-2 (PMC9873389; doi:10.1186/s12889-023-15075-2)
Supplement: Supplementary file 1 — Additional file 1: Table S1. Descriptive statistics of the health utility values, 2008–2020. Table S2. Multiple linear regression analyses on health utility values (Polled). [file 12889_2023_15075_MOESM1_ESM.pdf]

BMC Public Health

The Trend in Quality of Life of Chinese Population: Analysis based on Population Heath Surveys from 2008 to 2020

Supplementary Information

Dingyao Wang[1,2], Shitong Xie, PhD[1,3], Jing Wu, PhD[1,2,*], Bei Sun[4,**]

[1] School of Pharmaceutical Science and Technology, Tianjin University, Tianjin, China

[2] Center for Social Science Survey and Data, Tianjin University, Tianjin, China

[3] Department of Health Research Methods, Evidence, and Impact, McMaster University, Hamilton, Ontario, Canada

[4] Academy of Medical Engineering and Translational Medicine, Tianjin University, Tianjin Medical University Cancer Institute and Hospital, National Clinical Research Center for Cancer, Key Laboratory of Cancer Prevention and Therapy, Tianjin, Tianjin's Clinical Research Center for Cancer, Tianjin, China

[*] **Corresponding author:** Jing Wu, PhD, Professor, School of Pharmaceutical Science and Technology, Tianjin University, Tianjin, China, 300072. E-mail: jingwu@tju.edu.cn

[**] **Corresponding author:** Bei Sun, Academy of Medical Engineering and Translational Medicine, Tianjin University, Tianjin Medical University Cancer Institute and Hospital, National Clinical Research Center for Cancer, Key Laboratory of Cancer Prevention and Therapy, Tianjin, Tianjin's Clinical Research Center for Cancer, Tianjin, China, 300072. E-mail: sunpei003@sina.com

Table A1 Descriptive statistics of the health utility values, 2008-2020

	Health utility values		
	2008 (N=25,939)	2013 (N=22,138)	2020 (N=19,177)
**Excepted utility method**			
Mean[a]	0.948	0.942	0.939
Median	0.974	0.974	1
SD	0.102	0.103	0.168
SE	0.001	0.001	0.001
Range (Min, Max)	(-0.092, 0.974)	(-0.092, 0.974)	(-0.391, 1)
Kurtosis	40.862	35.598	25.896
Skewness	-5.631	-5.108	-4.417
**Original**			
Mean	0.967	0.958	0.939
Median	1	1	1
SD	0.122	0.125	0.168
SE	0.001	0.001	0.001
Range (Min, Max)	(-0.149, 1)	(-0.149, 1)	(-0.391, 1)
Kurtosis	31.211	26.014	25.896
Skewness	-4.922	-4.331	-4.417
**Monte Carlo simulation method**			
Mean[a]	0.948	0.942	0.939
Median	1	0.955	1
SD	0.111	0.111	0.168
SE	0.001	0.001	0.001
Range (Min, Max)	(-0.244, 1)	(-0.216, 1)	(-0.391, 1)
Kurtosis	32.611	29.008	25.896
Skewness	-4.803	-4.388	-4.417
**Most-likely probability method**			
Mean[a]	0.979	0.974	0.939
Median	1	1	1
SD	0.097	0.097	0.168
SE	0.001	0.001	0.001
Range (Min, Max)	(-0.158, 1)	(-0.158, 1)	(-0.391, 1)
Kurtosis	60.826	56.399	25.896
Skewness	-6.986	-6.605	-4.417
**DSU method**			
Mean[b]	0.959	0.946	0.902
Median	1	1	0.977
SD	0.157	0.164	0.178
SE	0.001	0.001	0.001
Range (Min, Max)	(-0.594, 1)	(-0.594, 1)	(-0.577, 0.989)
Kurtosis	39.137	31.482	24.735
Skewness	-5.526	-4.782	-4.025

Note: Significant differences were found in health utility values across the three waves of data by Kruskal-Wallis H tests ($p<0.001$).

[a] The EQ-5D-3L responses were mapped to EQ-5D-5L responses by the UK response mapping algorithm [28] and then converted to utility values for the mapped responses using corresponding method [29].

[b] The responses of respondents in 2020 elicited from the EQ-5D-5L were indirectly mapped to the EQ-5D-3L utility values by the DSU method [31].

Abbreviation: DSU, Decision Support Unit; Max, maximum value; Min, minimum value; NICE, the National Institute for Health and Care Excellence; SD, standard deviation; SE, standard error.

Table A2 Multiple linear regression analyses on health utility values (Polled)

Independent variables	Health utility values [a] (N=67,254)		
	β	95% CI	p value [*]
**Year (vs. 2008)**			
2013	-0.009	(-0.012, -0.007)	**<0.001**
2020	-0.010	(-0.012, -0.009)	**<0.001**
**Female (vs. Male)**	-0.001	(-0.002, 0.001)	0.510
**Han Chinese (vs. Others)**	-0.009	(-0.015, -0.002)	**0.009**
**Age group (vs. 18-29)**			
30-39	-0.003	(-0.005, -0.001)	**0.002**
40-49	-0.004	(-0.005, -0.001)	**0.001**
50-59	-0.004	(-0.006, -0.001)	**0.003**
60-69	-0.016	(-0.020, -0.013)	**<0.001**
≥ 70	-0.079	(-0.084, -0.073)	**<0.001**
**Commercial medical insurance (vs. Yes)**	-0.004	(-0.007, -0.002)	**0.001**
**Recipients of medical assistance (vs. No)**	-0.066	(-0.082, -0.051)	**<0.001**
**Marital status (vs. Married)**			
Unmarried	-0.010	(-0.013, -0.007)	**<0.001**
Widowed	-0.020	(-0.026, -0.014)	**<0.001**
Divorced	0.000	(-0.006, 0.006)	0.894
**Education (vs. College or above)**			
Primary or below	-0.022	(-0.026, -0.018)	**<0.001**
Junior high school	-0.010	(-0.012, -0.007)	**<0.001**
Senior high school	-0.004	(-0.007, -0.002)	**<0.001**
**Employment status (vs. Employed)**			
Retired	-0.005	(-0.008, -0.002)	**0.002**
Student	-0.001	(-0.004, 0.002)	0.473
Unemployed	-0.020	(-0.023, -0.018)	**<0.001**
**Hypertension (vs. No)**	-0.018	(-0.021, -0.015)	**<0.001**
**Diabetes (vs. No)**	-0.021	(-0.026, -0.016)	**<0.001**
**Other chronic diseases (vs. No)**	-0.055	(-0.060, -0.051)	**<0.001**
**Number of illnesses in 2 weeks (vs. 0)**			
1	-0.018	(-0.024, -0.012)	**<0.001**
2 or more	-0.031	(-0.044, -0.019)	**<0.001**
**Hospitalizations in 12 months (vs. No)**	-0.056	(-0.064, -0.048)	**<0.001**
**R^2**		0.170	

[*] The p-value with bold formatting represents significant in multivariable linear regression model at 0.05 level.

[a] The EQ-5D-3L responses were mapped to EQ-5D-5L responses by the UK response mapping algorithm [28] and then converted to utility values for the mapped responses using the excepted-utility method [29].

Abbreviation: 95% CI, 95% confidence interval.
